# Comparing the Experiences of Participants With Mild Cognitive Impairment and Mild Dementia During an Oral Health Intervention

**DOI:** 10.1093/geroni/igad123

**Published:** 2023-10-26

**Authors:** Bianca Shieu, Chiao-Hsin Teng, Ya-Ning Chan, Youngmin Cho, Cassandra Dictus, Jing Wang, Anna S Beeber, Ashley Bryant, Bei Wu, Brenda L Plassman, Ruth A Anderson

**Affiliations:** School of Nursing, UT Health San Antonio, San Antonio, Texas, USA; School of Nursing, University of North Carolina at Chapel Hill, Chapel Hill, North Carolina, USA; Department of Population Health Sciences, Duke University, Durham, North Carolina, USA; School of Nursing, University of North Carolina at Chapel Hill, Chapel Hill, North Carolina, USA; School of Nursing, University of North Carolina at Chapel Hill, Chapel Hill, North Carolina, USA; College of Health and Human Services, University of New Hampshire, Durham, New Hampshire, USA; School of Nursing, Johns Hopkins University, Baltimore, Maryland, USA; School of Nursing, University of North Carolina at Chapel Hill, Chapel Hill, North Carolina, USA; Rory Meyers College of Nursing, New York University, New York, USA; Department of Psychiatry and Behavioral Sciences, Duke University, Durham, North Carolina, USA; School of Nursing, Duke University, Durham, North Carolina, USA

**Keywords:** Adaptive leadership framework for chronic illness, Alzheimer’s disease, Community settings, Oral care intervention

## Abstract

**Background and Objectives:**

Cognitive impairment is associated with poor oral health outcomes. Oral hygiene tasks are an essential target of interventions aiming to improve oral health for older adults with cognitive impairment. We aimed to examine whether experiences in an oral health intervention based on the Adaptive Leadership Framework for Chronic Illness differed between individuals with mild cognitive impairment (MCI) or mild dementia (MD) and their respective care partners.

**Research Design and Methods:**

This was a secondary analysis using directed content analysis and then an interpretive-description approach to analyze the data from a theory-driven intervention study. We included 10 people with MCI and their care partners (*n* = 20) and 8 people with MD and their care partners (*n* = 16) in the treatment arm of the intervention. For each participant, we analyzed audio recordings of 4 intervention coaching sessions, each ranging between 30 and 45 min. We managed the data and coding using ATLAS.TI software.

**Results:**

Participants in both the MCI and MD groups experienced similar challenges in adapting to changes in oral hygiene techniques, and both groups worked on learning new oral hygiene techniques taught by the dental hygienist and meeting individualized goals developed with their care partner, interventionist, and hygienist. On the other hand, there were subtle differences in technical challenges between participants in MCI and MD groups; participants in the MCI group reacted more actively to dental hygienist suggestions than the MD group.

**Discussion and Implications:**

Study findings provide information about how researchers and clinicians might tailor interventions to meet the learning needs of individuals and care partners in each group.

**Translational Significance:** Our study compared participants in mild cognitive impairment (MCI) and mild dementia (MD) groups and found they experienced similar challenges in adapting to changes in oral hygiene techniques. Both groups learned new oral hygiene techniques and met individualized goals developed with their care partner, interventionist, and hygienist. Participants in MCI and MD can learn new oral techniques, and they adapt and continue to learn. However, clinicians need to use tailored approaches based on the level of cognitive impairment of the patient. Interventions guided by the Adaptive Leadership Framework for Chronic Illness framework are an excellent approach to facilitating personal care among the aging population.

As the population ages internationally, the number of people with dementia is estimated to nearly triple to more than 152 million worldwide by 2050 (Alzheimer’s Association, 2021). An estimated 6.2 million Americans aged 65 and older were living with Alzheimer’s dementia in 2021, which is more than one in nine people (11.3%) in this age range with the disease (Alzheimer’s Association, 2021). Roughly 22% of the 62 million Americans who are aged 65 and older in 2023 have mild cognitive impairment (MCI) due to any cause (Alzheimer’s Association, 2023). Of those with MCI, about 15% develop dementia after 2 years (Petersen et al., 2018). Cognitive impairment is associated with poor oral health outcomes (Demmer et al., 2020; Iwasaki et al., 2019; Kang et al., 2020; Qi et al., 2021). These outcomes include lower quality of life due to oral pain (Delwel et al., 2017) and malnutrition (Thoniazzo et al., 2018), poor general health (Griffin et al., 2012), and specific health conditions, including pneumonia (van der Maarel-Wierink et al., 2013), diabetes (Luo, 2015), and cardiovascular disease (Leng et al., 2015). Preventing or slowing oral diseases (e.g., tooth decay and periodontitis) is possible through regular and thorough oral hygiene practices and treatment (Davies et al., 2002; Grender et al., 2013). Yet one study reported that only 44% of community-dwelling individuals with mild to moderate dementia brush their teeth at least twice daily compared to 72% of those with normal cognition (Lee et al., 2016). Therefore, oral hygiene tasks are an essential target of interventions aiming to improve oral health for older adults with cognitive impairment.

As interventions are developed for individuals with cognitive impairment, it is important to consider targeting early stages of these disorders, such as MCI and mild dementia (MD), when both behavior change and oral disease prevention are more likely (Bahar-Fuchs et al., 2013; Wu et al., 2008). Though cognitive impairment is more severe in MD than MCI, activities of daily living, like toothbrushing, are long-practiced routine skills that are part of procedural memory and are typically preserved in both MCI and MD (Matthews et al., 2008; Zanetti et al., 1997). People with MCI and MD show differences in related health outcomes. For instance, our own study showed that the oral health-related quality of life was significantly different across the spectrum of cognitive function, with the highest scores among normal cognitive function group, followed by the MCI group, and the lowest scores for the mild dementia group (Lee et al., 2013). Given differences in cognitive functioning, people with MCI or MD may have different experiences in response to an oral health intervention; however, this had not been explicitly researched.

Our recent pilot study of a care partner-assisted intervention to improve oral health for community-dwelling older adults with cognitive impairment included participants with MCI and MD (Anderson et al., 2019; Wu et al., 2021). In this intervention, developed using the Adaptive Leadership Framework for Chronic Illness (ALFCI; Anderson et al., 2015), we proposed that self-managed chronic illnesses often include both technical components traditionally taught by the healthcare team and behavior adaptations completed in collaboration with care partners. Because this study included participant groups with both MCI and MD, it provided an opportunity to examine how cognitive impairment levels related to different experiences during the ALFCI-based oral hygiene intervention. Additionally, the intent is that adaptive strategies used during the ALFCI-based interventions, such as cueing and optimized communication, can be applied to other challenges people with MCI and MD and their care partners may face. Thus, the intervention may improve their generalized ability to adapt to various day-to-day challenges that might otherwise cause them distress.

The purpose of this study was to describe and compare the experiences of people with MCI and MD during a coaching intervention in which care partners were taught to facilitate proper oral hygiene among individuals with MCI or MD. Specifically, we aimed to identify commonalities and differences among people with MCI or MD in challenges and strategies used during an oral health intervention.

## Method

### Design

**Figure 1.**
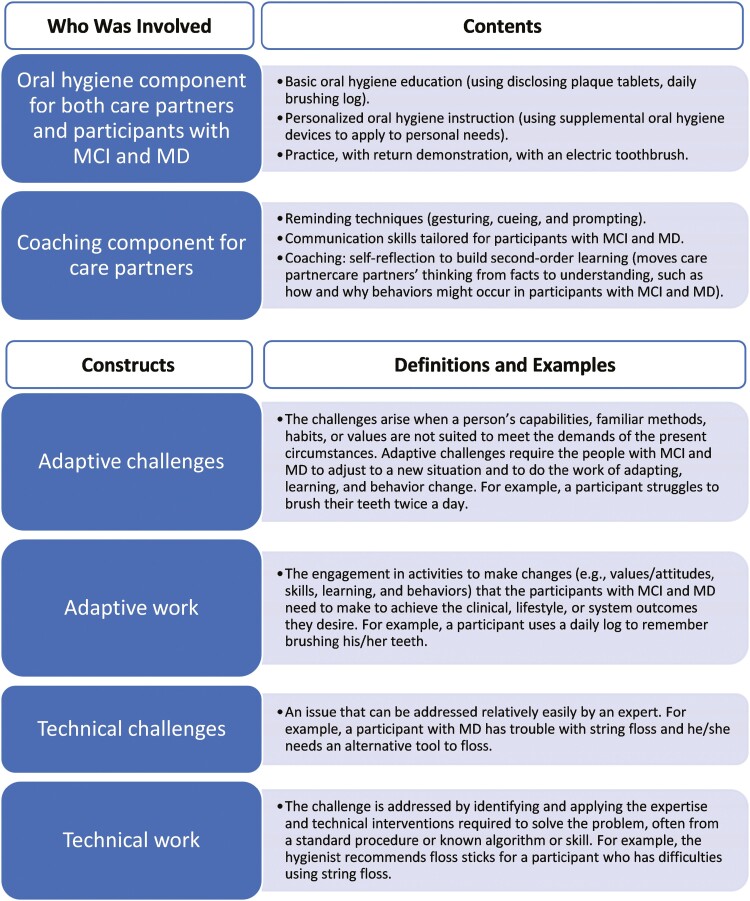
The Adaptive Leadership Framework for Chronic Illness (ALFCI) guided intervention. MCI = mild cognitive impairment; MD = mild dementia.

This was a secondary analysis using directed content analysis and then an interpretive-description approach to analyze the data from a theory-driven intervention study (Thorne et al., 1997). We begin with directed content analysis using the four a priori codes: adaptive challenge, adaptive work, technical challenge, and technical work (Figure 1; Hsieh & Shannon, 2005). We used a priori codes because the intervention study was driven by the ALFCI framework. In addition, it allowed us to understand the adaptive challenge/work that can only be done by the person with the illness and/or his or her family as well as the technical challenges/work that need assistance from healthcare providers. Then we identified themes within each of these four broad categories of data using an interpretive-description approach (Thorne, 2016). We used an interpretive-description approach because it allowed us to use established framework (ALFCI) as a lens to understand the complex phenomena that how MCI and MD encountered challenges similarly and differently and then illuminate implications for future education among people with MCI and MD (Thompson Burdine et al., 2021). We followed the guideline of *Standards for Reporting Qualitative Research* to structure our study (O’Brien et al., 2014). This study was approved by the Institutional Review Boards at Duke University and the University of North Carolina at Chapel Hill.

### Brief Description of Intervention

The intervention, guided by ALFCI, was designed to improve the oral hygiene behaviors of participants with MCI and MD and help their care partners become leaders who assist participants with MCI and MD in engaging in the oral hygiene activities (Wu et al., 2021). The intervention emphasized the collaboration of participants with MCI or MD with their care partners (Wu et al., 2022). The intervention included (1) an oral hygiene component to help participants and care partners learn proper oral hygiene knowledge and technique and (2) a care partner component to learn adaptive strategies to use in a tailored manner for participants with MCI and MD (Figure 1). The intervention included four coaching sessions at (1) baseline (home visit), (2) Week 4 (by phone), (3) Week 8 (by phone), and (4) Week 12 (home visit). Each session lasted about 30–45 min. In the sessions with care partners, the interventionist, who was a nurse with experience in home-based dementia care and caregiver support, used guided questions/prompts to develop care partner’s goals for using communication strategies such as memory prompts, gesturing, and verbal and visual cues. Some examples of guided prompts are: “How has [insert participant’s name] been doing with the new toothbrush and brushing techniques since our last visit?”; “When we talked last time, you set some goals to [insert the highest priority goal from the list]. How have you done with [insert correct word such as using the toothbrush, amount of time spent brushing, flossing as relevant to the goal list created by the participant and care partner].” Participants also joined the last portion of the coaching session that was completed by phone to discuss their SMART goals (Specific, Measurable, Action-oriented, Reasonable, and Timely) related to oral hygiene behaviors. The study dental hygienist joined home visit coaching sessions (baseline and 3 months) to engage in teaching oral hygiene techniques, and collaborate with the interventionist, participant, and care partner on a joint plan of action.

### Data Source

The study included 10 people with MCI and their care partners (*n* = 20) and eight people with MD and their care partners (*n* = 16) in the treatment arm of the intervention (Wu et al., 2021). People with MCI or MD in our study are referred to as “participants” and the person living with them and supporting them during the intervention as a “care partner.” Data in this study were verbatim transcripts of audio recordings of the four coaching sessions with care partners and participants in the intervention group. Transcripts were deidentified and checked for accuracy (by reviewing transcripts while listening to the original recordings).

### Data Analysis

Prior to coding, each transcript was verified by two coders and analyzed by three coders. We first used directed content analysis with ALFCI constructs as a priori *codes* to code transcripts broadly (Figure 1; Hsieh & Shannon, 2005). We managed the data and coding using ATLAS.TI software. The coding team included seven coders who are current graduate students with advanced training in qualitative methods and a senior researcher lead. Seven coders and the lead researcher read several transcripts and coded the same transcripts until everyone reached a consensus and consistency in coding. The entire team used the a priori codes of the ALFCI and definitions as the main themes: (1) adaptive challenges, (2) adaptive work, (3) technical challenges, and (4) technical work (Figure 1; Anderson et al., 2015).

Then, within each of these categories of data, we used an interpretive-description approach to analyze quotes inductively to develop subthemes in each construct (Thorne, 2016). With guidance from senior researchers, the analytic team developed an inductive coding process in which six coders (one coder left) were divided into two groups. In each group, every coder first rereads the relevant quotes under each theme (e.g., adaptive challenges) and grouped quotes that are related thematically. Each coder proposed their own potential subtheme topics in the within-group meeting and later finalized a single list of subthemes on which the group agreed. Finally, the two groups met together (i.e., between-group meeting) to compare the findings between participants with cognitive impairments (MCI [*n* = 10] and MD [*n* = 8]). Throughout this phase, the coders again consulted with senior researchers when coding clarification was needed or to resolve coding disagreements. After the two coding groups examined the differences and similarities among challenges and adaptive strategies used, a lead coder in each group presented the findings to senior qualitative researchers who served as external checkers. Memos were used to record the coders’ thought processes and decisions to keep an audit trail to ensure rigor in qualitative research (Lincoln & Guba, 1985).

We included any prominent subthemes that were not directly related to oral health but were deemed relevant to the intervention delivery of the adaptive behaviors being learned. We surmised that nonoral health challenges raised by participants or care partners might indirectly affect the success of the intervention and shed light on the experiences of participants and caregivers during a behavior-change intervention. For example, if the overall relationship between the participant and the care partner is tense, they may not have a good quality of collaborative work regarding oral healthcare.

## Results

Table 1 presents the sample characteristics of 10 pairs in the MCI group and eight pairs in the MD group. The average age of the MCI and MD groups were 72 and 73.4, respectively. The MCI group had an average baseline score of 22.6 on the Montreal Cognitive Assessment, whereas the MD group had an average score of 16.38. In both subgroups, the majority of individuals had their spouse as their care partner.

**Table 1.** Sample Characteristics of MCI and MD Groups

Characteristic	MCI group		MD group	
	Participant (*n* = 10)	Care partner (*n* = 10)	Participant (*n* = 8)	Care partner (*n* = 8)
	Mean (*SD*) or frequency [%]			
Age (years)	72 (6.32)	66.2 (9.17)	73.4 (5.66)	68.1 (15.2)
Gender (male)	8 [80]	3 [30]	4 [50]	4 [50]
Race/ethnicity				
White	9 [90]	9 [90]	6 [75]	6 [75]
Black	1 [10]	1 [10]	1 [12.5]	1 [12.5]
Asian	0	0	1 [12.5]	1 [12.5]
Baseline MoCA	22.6 (3.92)	—	16.38 (3.46)	—
Education years	17.1 (0.99)	15.5 (2.51)	9.6 (2.33)	8.25 (2.05)
Care partner relationship				
Spouse	—	7 [70]	—	7 [87.5]
Children	—	1 [10]	—	1 [12.5]
Others	—	2 [20]	—	0

*Notes*: MCI = mild cognitive impairment; MD = mild dementia; MoCA = Montreal Cognitive Assessment; *SD* = standard deviation.

The findings below are presented under the four main theory-directed themes (adaptive challenges, adaptive work, technical challenges, and technical work) for the participant groups (MCI and MD). The bolded words under each of the theory-directed themes represent subthemes. Because the recorded coaching sessions were conducted primarily with the care partners, most of the selected quotes were from care partners as they shared their observations and perspectives about the participants’ experiences during the intervention.

### Adaptive Challenge

Participants in both MCI and MD groups experienced similar challenges in adapting to changes in **oral hygiene techniques and tools**, especially in following the specific toothbrushing instructions (e.g., frequency) and using the new electric toothbrush. One care partner (wife) stated a participant with MCI did not understand instructions well or did not remember when/how often he was supposed to do oral hygiene: “It was more about changing the direction of how he makes the brush go down. And his own way of managing his oral health is that sometimes he doesn’t think at the end of the day he needs to brush his teeth and he just rinses with mouthwash.” In the MD group, one care partner (daughter) stated that the participant with MD had difficulty in acquiring the new toothbrushing technique: “You know, in terms of like he turns it like the wrong direction. So, and I keep just showing him, look, you have to do this way, but he inadvertently goes back to the way he was doing it.”

Additionally, participants in both groups experienced adaptive challenges related to **cognitive and functional changes**, such as completing tasks more slowly, making mistakes, and being forgetful during the intervention. In the MCI group, for instance, one participant forgot and did not use the plaque-disclosing tablet, “I guess I could start going back [to use the plaque disclosing tablets]. I put them [the tablets] in the drawer and I really forgot to take them out.”

Participants in the MD group indicated more pronounced adaptive challenges related to cognitive and functional changes, such as understanding and learning the toothbrushing techniques. As one care partner (wife) from the MD group stated, “He forgets. I’m seeing more and more forgetfulness from him. … he is using the brush, but I still don’t think that he’s really doing it correctly.”

The three subthemes below—relationship issues, emotional distress, and loss of independence—were adaptive challenges that were not always directly related to oral health. However, these reoccurring topics are likely to interfere with participants adaptive challenges in oral health.

Conflicts arose between participants and care partners when working together in both groups; however, we saw nuanced differences between the groups in how the **relationship issues** presented. Participants in the MCI group tended to **argue with their care partners**. For example, one care partner (wife) stated, “as I’ve gotten older … I don’t have patience. I find, ‘Why didn’t you take out the trash? (whispering)’ … I’m saying, it’s a problem. And of course, he gets very defensive because of the way I said it.” On the other hand, participants in the MD group showed rejection of care partners’ advice or ended a conversation by **ignoring** care partners. As one care partner (husband) stated, “he acted as though he resented me watching him brush his teeth or brushing along with him and he wouldn’t listen to me when I would try to suggest that he do it a certain way, and he just wouldn’t watch me. It agitated him.”

Participants in the MCI group demonstrated **emotional distress** but that was not found in the MD groups. In the MCI group, participants had negative feelings and mood fluctuations when encountering changes in cognition, priorities, and role in life. For example, one care partner (husband) stated, “I would say that she gets very frustrated when she can’t remember something.”

On the other hand, care partners in the MD group reported concerns that participants **lost independence in daily life**, and this was not found in the MCI group. They were no longer able to act entirely independently in accomplishing basic self-care or day-to-day tasks such as bathing, cooking, grocery shopping, or personal finances due to safety concerns or potential issues (e.g., incontinence, physical injury when using power tools, or car accident).

### Adaptive Work

Participants in both MCI and MD groups worked on **learning new oral hygiene techniques** taught by the dental hygienist and tried to meet individualized goals developed with their care partner, interventionist, and hygienist. They either **practiced by themselves or collaborated with care partners** who facilitated performance. For example, one participant from the MCI group stated, “We started that originally, doing it [working together on toothbrushing in the bathroom] every time … kind of facing each other … and it’s hard for me to keep track of what I’m doing … I was more concerned about doing it correctly than anything. But I think that helped get me started and once I got started you know, we basically do it every few days. Still face to face [with care partner], but I generally can, I think I do it correctly.” Similarly, another care partner (wife) in the MD group mentioned, “He generally gets up and he’ll go and brush his teeth like he’s supposed to, and even sometimes if he forgets to after lunch, and it, a little bit of time goes, and he’ll say, ‘Oh, I forgot to brush my teeth,’ and he’ll go on in there and do it, and he’s still pretty much able to do on his own at this point.”

In both MCI and MD groups, **visual and verbal cueing strategies** were used to facilitate their adaptive work. For example, one care partner (daughter) from the MCI group stated, “They [log notes] are working out pretty good, my father’s trying to stay on track with his … the logs … (visual cueing).” Another care partner (husband) from the MD group stated, “He might not remember [to use the plaque disclosing tablet], so I’m going to try to catch him before he gets started (verbal cueing).”

We also saw some differences related to cueing. Some participants in the MD group also responded to **tactile cues** to adapt a new oral hygiene technique, while this cueing technique was not seen often in the MCI group. One care partner (daughter) from the MD group said, “I try to, use my hand to make her hand relax and let it do the work.”

Participants in the MCI group **adapted with a new attitude** related to oral care intervention and changes in daily routine. This was not seen in the MD group. For instance, one participant started learning to accept using the new toothbrush.

Care partner (girlfriend): [Participant] didn’t like the toothbrush initially anyway, but he had no choice. So, he said he’s gotten used to it, it’s not as bad now as it was before. It’s just that the vibration is just so much more than what we’re used to. And initially, I did have to coach him on the speed, but he’s much better at that now. I just simply said to him, “Don’t forget honey, slow it down, go a little more slowly, now go back over that area more slowly,” and then just when he did it correctly, I said, “Yup. That’s perfect.” So, it’s just really just staying on him.

Another care partner in the MCI group observed the participant showed acceptance of the life changes that needed to be made as noted in the following quote:

Care Partner (wife): I think what we’re doing well is that we are … I’m reaching out and he’s accepting some of these … new connections. You know, doing family sports for one, reaching out more to our friends and neighbors in a different way once we, once he made the decision to share with close friends what is going on. So, I think that part we’re doing well.

### Technical Challenge

Participants in both the MCI and MD groups **required additional information or instructions** from the dental hygienist on using oral hygiene tools (e.g., an electric toothbrush or floss) or employing proper oral hygiene techniques. For example, one participant in the MD group encountered drooling while using an electric toothbrush and the dental hygienist gave him additional instructions: “The only issue is sometimes I got drool coming out of my mouth, but now [I follow dental hygienist instruction] and I will get a hold of a toothbrush and start c-cleaning away with my teeth and … I am used to it now. Much more so than in the early stages.” Another participant in the MCI group had difficulty brushing certain parts of the distal surface or mesial surface of teeth. As one stated, “I’m still struggling. I’m still having a little trouble doing the back of my teeth. You know, trying to adjust the … brush head to get the … full brushing. But that’s why it takes me about four minutes, rather than two.”

Additionally, some participants in both groups had **preexisting physical conditions** that made it difficult for them to use the toothbrushes and/or floss. One participant in the MCI group stated. “I had broken my arm last June. so, I don’t have a lot of use of my hand. I still don’t. And I never will have a good of use of [the new electric toothbrush].” Similarly, another care partner (husband) in the MD group stated, “The biggest reason that she has trouble flossing is the one finger that she has [arthritis] problems with.”

There were subtle differences in the process of figuring out the technical challenges between MCI and MD group participants. For example, some participants from the MCI group **recognized their own challenges** by themselves during the coaching session. They mentioned they had difficulty brushing certain parts of their teeth to the dental hygienist and asked for possible solutions, whereas this was not reported in the MD group. Also, participants in the MCI group **actively engaged in the coaching sessions** to seek advice or solutions from the dental hygienist due to physical limitations and lack of knowledge of oral hygiene tools. For example, one participant could not decide the proper amount of toothpaste to use and stated, “I tend to slobber a lot so I have to stop slightly and spit it out, but it seems to create more liquids and more saliva. … so I am not sure how much toothpaste I should use.”

### Technical Work

Participants in both MCI and MD groups were **advised on oral hygiene techniques** by the dental hygienist. For example, one participant in the MCI group who received information about the correct way of brushing the teeth from the dental hygienist, stated, “she gave some good clues about how to do it. … I noticed that it’s rotating your arm, the way she was doing. It wasn’t hard to do at all.” Similarly, participants in the MD group were advised on techniques they lacked or were provided with individualized solutions to solve problems encountered during oral care, such as the interventionist giving additional instruction to one participant who drooled when using a new electric toothbrush.

Participants in both the MCI and MD groups **received recommendations regarding alternative or additional tools** to address a specific problem that could not be adequately addressed with the then-currently available gear. For example, fluoride toothpaste for decayed teeth, a smaller and nylon-coated toothbrush for an implant, floss picks, and smaller floss for tight spaces between teeth, especially when participants could not use string floss, as was sometimes the case. As one care partner (daughter) in the MCI group stated, “We had bought everything on the list that she [the dental hygienist] recommended.” Another care partner (husband) from the MD group stated, “I think he’s doing it a little bit better than I am because [the dental hygienist] brought him some little things [floss picks] to use. And I think he’s been using it more than I am.”

However, compared to the participants and care partners in the MD group, those in the MCI group discussed more technical challenges outside of oral hygiene care and how to resolve them during the sessions. They also **reacted more actively to dental hygienist suggestions** than the MD group. For example, the MCI group provided feedback after putting the additional oral hygiene instructions into practice from the dental hygienist, whereas this was shown less frequently in the MD group.

## Discussion

This is an intervention study using the ALFCI because we proposed that self-managed chronic illnesses often include both technical components, traditionally taught by the healthcare team, and behavior adaptations, completed in collaboration with care partners who is developed in the intervention to assume an adaptive leadership role. Under ALFCI, four constructs were examined and reported in this paper: adaptive challenges, adaptive work, technical challenges, and technical work, respectively (Figure 1). Furthermore, this framework has been used in other populations such as treatment for chronic hepatitis C (Bailey et al., 2021), intensive care unit (ICU) end-of-life care (Adams et al., 2013), and fatigue after stroke (Teng et al., 2023).

Using the ALFCI as a guide, we described and compared the experiences of people with MCI and MD who participated in the care partner-assisted intervention as follows: (1) Participants in both MCI and MD groups experienced similar *adaptive challenges* in using the new electric toothbrushes and following the toothbrushing instructions, but only participants in the MD group demonstrated more profound adaptive challenges related to cognitive and functional changes, such as understanding and learning the toothbrushing techniques. (2) Participants in both MCI and MD groups performed *adaptive work* on learning new oral hygiene techniques and meeting the individualized goals developed with their care partner, interventionist, and hygienist; however, only participants with MD benefited from tactile cues to adapt to new oral hygiene techniques. (3) For *technical challenges*, participants in both MCI and MD groups required additional information or instructions on using oral hygiene tools (e.g., the electric toothbrush or floss) or employing proper oral hygiene techniques; but only participants with MCI recognized their own challenges. (4) Related to *technical work*, participants in both MCI and MD groups received advice from a dental hygienist on how to apply better hygiene techniques, whereas only participants in the MCI group presented a proactive attitude in response to dental hygienist suggestions/advice (see Table 2).

**Table 2.** The Comparison of Participants in the MCI and MD Groups According to the Themes

Theme	Commonalities among both MCI and MD groups	Differences between MCI and MD groups	
		MCI	MD
Adaptive challenge	• Experience challenges in adapting oral hygiene behavior and tools. • Become slower, more forgetful, and make more mistakes.	• Work on oral care intervention with care partners and argue with them when conflicts happen. • Present emotional distress.	• Work on oral care intervention with care partners and ignore/refuse them when conflicts happen. • Present difficulties in comprehension and learning toothbrushing techniques. • Experience loss of independence in life.
Adaptive work	• Work on oral hygiene techniques and goal setting. • Practice alone or join with care partners. • Use of visual and verbal cues.	• Adapted to changes with a new attitude.	• Use of tactile cues.
Technical challenge	• Have difficulty using the oral hygiene tools. • Correction and instructions were required to improve oral hygiene technique. • Additional advice/instruction was required due to preexisting health conditions.	• Recognize their own challenges. • Be proactive in their performance and seek advice from the dental hygienist.	• Passively evaluate their performance and recognize their challenges during the interview.
Technical work	• Received oral hygiene instructions related to techniques and tools from the dental hygienist and interventionist.	• Active attitude in response to dental hygienist suggestions.	• Passive attitude in response to dental hygienist suggestions.

*Notes*: MCI = mild cognitive impairment; MD = mild dementia.

### Comparison of Findings With Previous Studies

Previous studies primarily focused on improving oral hygiene among older residents with dementia in nursing homes (Sloane et al., 2013; Volk et al., 2020; Weintraub et al., 2018). Weintraub et al. (2018) and Volk et al. (2020) provided didactic and hands‐on training for nursing staff to attend to residents’ oral hygiene and denture care. Results showed that training nursing home staff to provide a person‐centered, evidence‐based mouth care program for older residents with dementia can significantly improve their oral hygiene outcomes (Sloane et al., 2013). Furthermore, our findings add new insights to a previously published article (Anderson et al., 2019) by comparing the differences and commonalities between MCI and MD groups during coaching sessions in community-based settings. Existing studies have found that participants with MCI often encounter personality and behavior changes such as irritability, depression, and anxiety (Caselli et al., 2018). Furthermore, social behavior changes can lead to potential disruption in interpersonal relationships (Caselli et al., 2018; Henry et al., 2012). Findings of these prior studies align well with our study as we noted that participants with MCI tended to argue with their care partners and described more emotional distress than participants in the MD group. Participants with MCI appeared to benefit from the coaching sessions beyond oral health outcomes, such as resolving other challenges in daily living and learning adaptive strategies; this learning was seen less among participants with MD. On the other hand, participants with MD were more passive; they rarely asked dental hygienists for advice or discussed challenges beyond oral hygiene care. They often rejected care partners’ facilitation or ended a conversation by ignoring care partners. As described by care partners, participants with MD had lower levels of independence in everyday lives and faced more challenges in daily conversations than participants with MCI. The progressive cognitive and functional decline experienced by those with MD may have affected the ease with which they learned new oral care techniques.

### Implications for Future Interventions

Overall, the findings suggest that tailored strategies are needed, based on cognitive severity and other functional capabilities, to facilitate the care partner’s leadership in addressing challenges. For example, participants in both the MCI and MD groups experienced challenges in adapting oral hygiene behavior and tools, as well as becoming forgetful and making errors. Therefore, it is important to continue using constructive communication strategies that have been shown to be helpful with those with cognitive impairment (Haberstroh et al., 2011).

Participants with MCI demonstrated emotional distress and tended to argue with care partners when working on oral care intervention. Therefore, this suggests that interventions may benefit from providing emotional support to maximize the effectiveness/adherence of the intervention as other studies have shown that emotional support may be associated with better cognition among older adults without dementia (Kim et al., 2020). Additionally, participants with MCI were able to present a proactive attitude in response to dental hygienist suggestions. As a result, participants with MCI can be provided with a more self-directed style of intervention (Gureckis et al., 2012).

In contrast, participants in the MD group were facing challenges in daily conversation and tended to ignore care partners when conflict arose. Consequently, approaching the care partners with tailored education and communication strategies may help them assist participants with toothbrushing techniques (Lago et al., 2017). Furthermore, participants in the MD group often presented difficulties in comprehension and learning new toothbrushing techniques, as well as a less proactive attitude. Therefore, participants in an MD group may need a guided style of intervention, sensory-focused approaches, and more feedback from the hygienists and care partners to recognize their technical challenges (Legere et al., 2018).

### Strengths and Limitations

Our study had a small sample size; however, our qualitative comparisons provide a foundation to identify commonalities and differences among people with MCI or MD in challenges and strategies used during an oral health intervention as well as to generate new intervention approaches to test in future research and to guide the design of future interventions. Additionally, Because the coaching intervention primarily targeted change in the care partner behavior, the bulk of the coaching sessions were spent with them, and the participants were engaged at the end to help set oral health goals and review progress. Thus, the data are predominately from the care partner perspectives with limited input from participants. The findings from this unique study will allow us to conduct a future randomized controlled trial to compare the differences and similarities of participants with MCI and MD in community-dwelling settings and their response to a care partner-assisted intervention. Furthermore, while this is the first application of ALFCI to oral hygiene behaviors among community-dwelling people with cognitive impairment, ALFCI has been applied to a variety of other conditions, including treatment for chronic hepatitis C (Bailey et al., 2021), ICU end-of-life care (Adams et al., 2013), and fatigue after stroke (Teng et al., 2023). Thus, this ALFCI provides a comprehensive way to learn about MCI and MD individually and collaboratively with care partners, interventionists, and hygienists.

### Conclusions and Implications

Participants with MCI or MD experienced similar challenges in adapting to changes in oral hygiene techniques/tools and benefited from this home-based intervention led by care partners who facilitated and supported their adaptive work. One of the innovations of the current study was to compare the similarities and differences between the MCI and MD groups regarding their challenges and responses, which provides implications for tailoring future interventions. Further research is needed to explore the leadership capacity of care partners between both groups, how participants’ cognitive condition affects vulnerability to emotional distress, and how researchers can incorporate our findings in developing supportive strategies to meet the needs of people with MCI or MD.
